# Profiling of miRNAs in porcine Sertoli cells

**DOI:** 10.1186/s40104-020-00487-6

**Published:** 2020-08-17

**Authors:** Xiaoxu Chen, Yi Zheng, Xueliang Li, Qiang Gao, Tongying Feng, Pengfei Zhang, Mingzhi Liao, Xiu’e Tian, Hongzhao Lu, Wenxian Zeng

**Affiliations:** 1 College of Biological Science and Engineering, Shaanxi University of Technology, Hanzhong, 723001 China; 2 Key Laboratory of Animal Genetics, Breeding and Reproduction of the Ministry of Agriculture, College of Animal Science and Technology, Northwest A&F University, Yangling, 712100 Shaanxi China; 3 College of Life Science, Northwest A&F University, Yangling, 712100 Shaanxi China

**Keywords:** MicroRNAs, Pig, Sertoli cell, Spermatogenesis, TRAF3

## Abstract

**Background:**

Sertoli cells (SCs) create a specialized environment to support and dictate spermatogenesis. MicroRNAs (miRNAs), a kind of ~ 22 nt small noncoding RNAs, have been reported to be highly abundant in mouse SCs and play critical roles in spermatogenesis. However, the miRNAs of porcine SCs remain largely unknown.

**Methods:**

We isolated porcine SCs and conducted small RNA sequencing. By comparing miRNAs in germ cells, we systematically analyzed the miRNA expression pattern of porcine SCs. We screened the highly enriched SC miRNAs and predicted their functions by Gene Ontology analysis. The dual luciferase assay was used to elucidate the regulation of tumor necrosis factor receptor (TNFR)-associated factor 3 (*TRAF3*) by *ssc-miR-149*.

**Results:**

The analysis showed that 18 miRNAs were highly expressed in SCs and 15 miRNAs were highly expressed in germ cells. These miRNAs were predicted to mediate SC and germ cell functions. In addition, *ssc-miR-149* played critical roles in SCs by targeting *TRAF3*.

**Conclusion:**

Our findings provide novel insights into the miRNA expression pattern and their regulatory roles of porcine SCs.

## Background

Male fertility depends on continuous spermatogenesis throughout the reproductive lifetime of males. Spermatogenesis is an orchestrated process that includes self-renewal and differentiation of spermatogonial stem cells (SSCs), meiosis of spermatocytes and spermiogenesis [1–4]. The processes of spermatogenesis are regulated precisely by the microenvironment or niche in the seminiferous tubules [5, 6]. Sertoli cells (SCs) are arguably the most important contributor of the niche to mediate self-renewal and differentiation of SSCs [7]. As for the meiosis and spermiogenesis, SCs play a central role in creation of a unique immune privilege environment [8]. Historically, the acquisition of immune privileged germ cells is attributed to the blood-testis barrier (BTB) that is composed of Sertoli cell-Sertoli cell junctions and Sertoli cell body [9]. SCs also express immuno-regulatory factors that actively suppress innate, humoral and cell-mediated immune regulation to maintain the immune privileged environment for spermatocytes and spermatids [10]. In the final stage of spermatogenesis, through processes of nuclear concentration, acrosome formation and tail formation, the round spermatid is transformed to the tadpole-like spermatozoa. Thus, SCs facilitate spermatozoa to release from SC surface and phagocytose the germ cell residual bodies [11]. Treatment of testis with benzalkonium chloride leads to depletion of SCs and further abnormal spermatogenesis [12]. The deficiencies of spermatogenesis can be rescued by the transplantation of healthy SCs, indicating the indispensable roles of SCs for spermatogenesis [12]. The function of SCs in supporting and dictating germ cell development triggers the question that how SCs maintain their own functions.

Two proliferation phases are taken place in porcine SCs. The first proliferation phase is from birth to one month of age, and the second phase is near puberty [13]. The roles of SCs switch from the regulation of testis formation to the maintenance of spermatogenesis during the puberty, which is termed maturation or differentiation of SCs [14]. The SC maturation that is mediated by follicle stimulating hormone, androgens, estrogens, thyroid hormones, retinoic acid, and growth factors [15] involves the loss of proliferative ability and formation of inter-Sertoli cell tight junctions [14]. The final number of Sertoli cells in adulthood is determined by proliferation of immature Sertoli cells. However, the regulatory mechanisms of porcine SC proliferation and maturation are not fully understood.

Recent studies have highlighted that microRNAs (miRNAs), a type of ~ 22 nt small noncoding RNA, are involved in the proliferation, maturation, apoptosis, autophagy, and pathogenesis of SCs in patients with Sertoli-cell-only syndrome [16]. The miRNAs that are abundant in human [17] and mouse [18] SCs are produced by Drosha and Dicer processing. In mouse SCs, the conditional depletion of Dicer 1 resulted in the defects of SC maturation and formation of cell junctions, suggesting that miRNAs play the critical roles in regulating SC functions [18–20]. In pigs, *ssc-miR-762* and *ssc-miR-1285* stimulated SC proliferation through distinct pathways in the cell cycle [21, 22]. In contrast, *ssc-miR-26a* and *ssc-miR-683* inhibited SC proliferation through targeting sperm associated antigen 1 (*SPAG1*) and p21 activated kinase 2 (*PAK2*), respectively [23, 24]. Although various function of miRNAs in SCs have been uncovered [16], the expression pattern and regulatory roles of miRNAs in porcine SCs remain unclear.

Pigs are not only as a livestock species that provide food source, but also as an ideal model bridges the gap between the rodents and humans in biomedicine. The proliferation and maturation of SCs are similar between pigs and humans [13]. Therefore, it would provide reference for biomedicine by analysis of porcine SCs miRNAs.

In this study, we isolated porcine SCs and established a comprehensive miRNA expression profile via small RNA-Seq. By comparing miRNA expression profiles from porcine SCs and germ cells that were reported previously, we identified the highly expressed miRNAs in SCs and predicted their potential roles. Our findings would provide novel insights into the regulatory mechanism of miRNAs in SCs and the correlations between SCs and spermatogenesis, which further provide potential insights into porcine reproduction and male infertility.

## Methods

### Animals

All experiments were approved by the Institutional Animal Care and Use Committee of the Northwest A&F University and performed in accordance with relevant guidelines and regulations (NWAFU Experimental Procedures 2018 No.318). The testes from 7 days old pigs were castrated under general anesthesia in the Bensun farm, Yangling. Three 5-month-old pigs were castrated under general anesthesia in the animal hospital of Northwest A&F University. After castration, the wounds were disinfected. Testes were stored in cold phosphate buffered saline (PBS) added with 2% of penicillin and streptomycin and brought to the laboratory immediately.

### Collection of SCs

The testes tissue from seven-day-old and five-month-old pigs was used for the isolation of immature and mature SCs, respectively. SCs were isolated as described previously with minor differences [25]. In brief, the testes were decapsulated and minced followed by 30 min digestion by collagenase Type IV (0.2% w/v; Invitrogen, USA) at 37 °C to collect seminiferous tubules. Then, 0.25% trypsin-EDTA (Hyclone, USA) was used to digest the seminiferous tubules to acquire single cell suspension. After removing of germ cells by differential plating, the SCs were cultured with DMEM/F12 containing 5% FBS and 1% penicillin and streptomycin at 37 °C. After three passages, SCs were harvested for the purity determination.

### Immunocytochemistry

To identify the purity of immature and mature SCs, cells were fixed with 0.4% paraformaldehyde (PFA) for 30 min at room temperature and washed with cold PBS for three times. Following permeabilizing using 0.4% triton-X 100, the cells were incubated with 1% BSA for 30 min to block the nonspecific reaction. Then, the mature SCs were incubated with rabbit anti-SOX9 (Abcam, Catologo numberi: ab76997) at a dilution with 1:400 and the immature SCs were incubated with rabbit anti-SOX9 at a dilution with 1:400 and mouse anti-AMH (Santa Cruz, Catologo numberi: sc-377,140) at a dilution with 1:200 overnight at 4 °C. Next day, the cells were washed with DPBS for three times and incubated with Alexa flour 488/594 conjugated donkey anti-rabbit IgG or Alexa flour 488 conjugated donkey anti-mouse IgG at 1:400 for 1 h at room temperature. After washed with DPBS, cell nucleus was labeled by DAPI (CWBIO, China) at a dilution with 1:400. The cell fluorescence was observed and photographed by a fluorescence microscope (Leica, Germany).

### Small RNA-Seq and analysis

Small RNA-Seq and analysis were performed as previous report [26]. Briefly, total RNA was used for adaptor ligation, first strand cDNA synthesis and PCR amplification to construct small RNA libraries. After sequencing of small RNA libraries by Illumina HiSeq 2000 sequencer, the acquired data were processed and mapped to reference sequence (Sus_scrofa.Sscrofa10.2). The differential expressed miRNAs between SCs and germ cells were analyzed with R DEGseq package [27].

The prediction of miRNA targets was conducted by miRanda and RNA hybrid. For miRanda, the alignment score of 160 or greater and an energy threshold of − 20.0 kcal/mol or less were consider as potential targets. For RNA hybrid, an energy threshold of − 20.0 kcal/mol or less were consider as potential targets. The gene ontology of miRNA targets was conducted by DAVID [28].

### Luciferase reporter assay

The dual-luciferase reporter vector psiCHECK2 was inserted with *TRAF3* 3’UTR at the site between NotI and XhoI. *TRAF3* mutant 3’UTR was amplified and cloned by homologous recombination according to the NovoRec® Plus PCR kit. Primers are listed in the Additional file 1.

PsiCHECK2 with targeted 3’UTR or mutant 3’UTR were co-transfected with miRNA mimics/NC into Hela cells using Lipofectamin2000 (Life technologies) according to the manufacturing protocols. The cells were collected for target validation assays using Dual Luciferase Assay System (Promega, USA) after 48-h culture.

### Quantitative RT-PCR (qRT-PCR)

Total RNA of SCs was extracted with Trizol. First strand cDNA was synthesized by using Transcriptor First Strand cDNA Synthesis Kit (Roche) according to the manufacturer’s protocol. The specific stem-looped primers were used for reverse transcription of specific miRNAs. qRT-PCR was performed with FastStart Essential DNA Green Master (Roche) using an IQ-5 (Bio-Rad). For the comparative Ct method (2 ^–ΔΔCT^), the relative expression level of genes and miRNAs was normalized to *GAPDH* and 5S RNA, respectively. Primers are listed in the Additional file 1.

### SCs cultures and challenges with lipopolysaccharide

After isolation, SCs were cultured with DMEM/F12 containing 5% FBS and 1% penicillin and streptomycin at 37 °C. SCs were counted and seeded in a six-well plate at a density of 100,000 cells/well. After 12 h culture, the cells were treated with lipopolysaccharide (LPS) from *Escherichia coli* O111:B4 (Sigma) for 3 h, 6 h, 12 h, 24 h, 48 h. The cells were washed by DPBS and re-suspended in Trizol for further analysis.

### Statistical analysis

Statistical analysis was conducted by SPSS V23.0. All values were presented as mean ± SEM, and *P* value less than 0.05 (*), 0.001 (**) was consider as significant difference.

## Results

### The composition of small RNAs in SCs

**Fig. 1:**
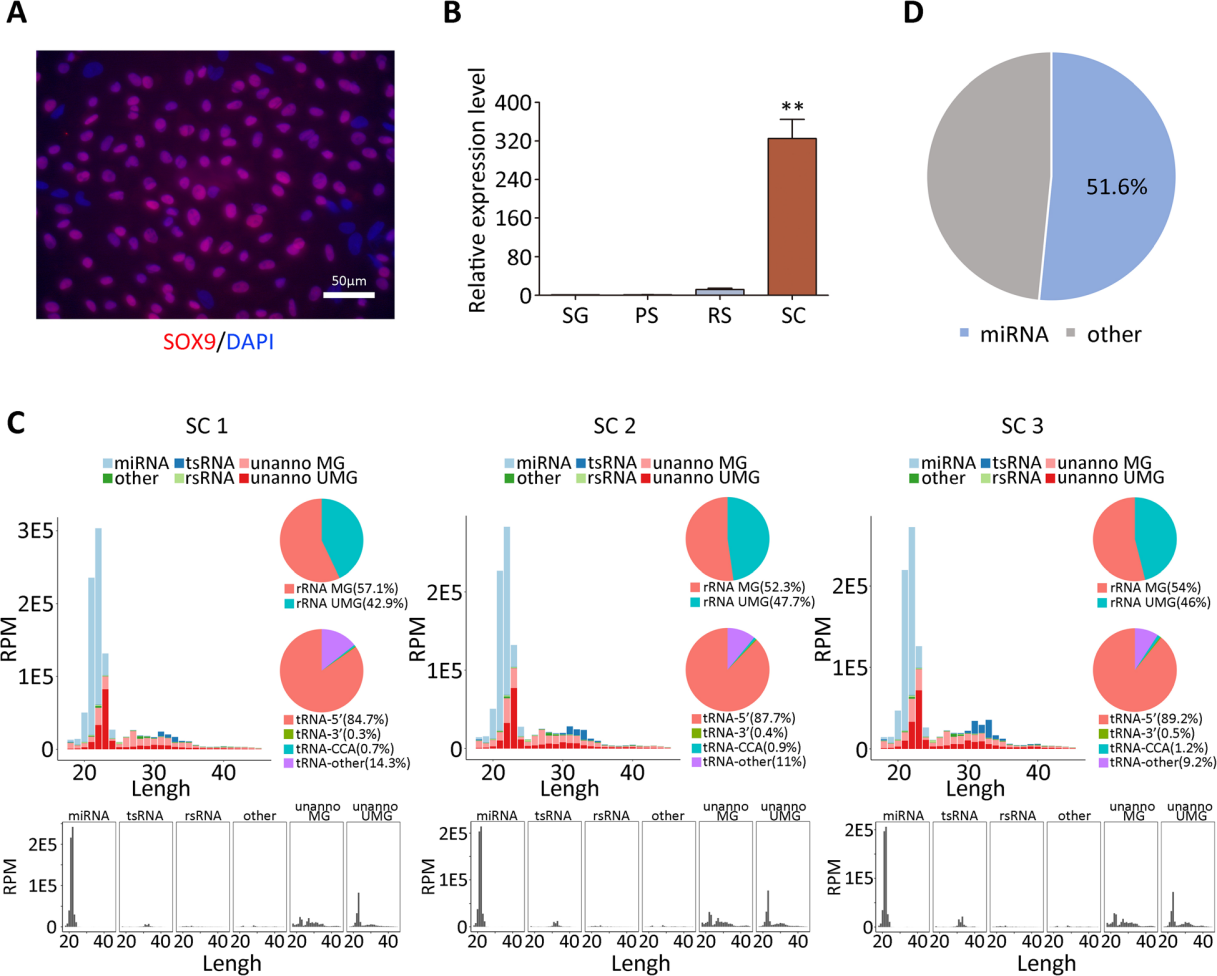
Identification of isolated Sertoli cells (SCs). (**A**) Immunocytochemistry analysis of SOX9 expression in SCs. (**B**) qRT-PCR analysis of SOX9 expression in SC compared with spermatogonia (SG), pachytene spermatocytes (PS) and round spermatids (RS). (**C**) Categories and length distribution of small RNAs in three SCs. (**D**) The percentage of miRNAs in small RNAs of SCs. Data were presented as the mean ± SEM, ** *P* < 0.01

To determine the purity of the isolated SCs, the expression of SC marker SOX9 was examined by the immunostaining. The 87.4% of the isolated mature SCs was positive for SOX9 (Fig. 1A). The expression level of *SOX9* in SCs was around 300 times higher than that in spermatogonia, pachytene spermatocytes and round spermatids (Fig. 1B), indicating that the purity of isolated SCs satisfied the requirements for small RNA-Seq. In Fig. 1C, the length distribution of small RNAs presented as two peaks. The first peak was mainly from 18 to 25 nt, which was higher than the second peak ranging from 26 to 35 nt (Fig. 1C). In 18 to 25 nt peak, miRNAs showed highest RPM level reflected by the height. Notably, miRNAs dominated (51.6%) the composition of small RNAs (Fig. 1D), suggesting the significance of miRNAs in SCs. In addition, the small RNAs derived from tRNA and rRNA distributed from 26 to 35 nt peak (Fig. 1C), indicating the potential roles of these two small RNAs in SCs.

### Analysis of miRNA expression in SCs and germ cells

**Fig. 2:**
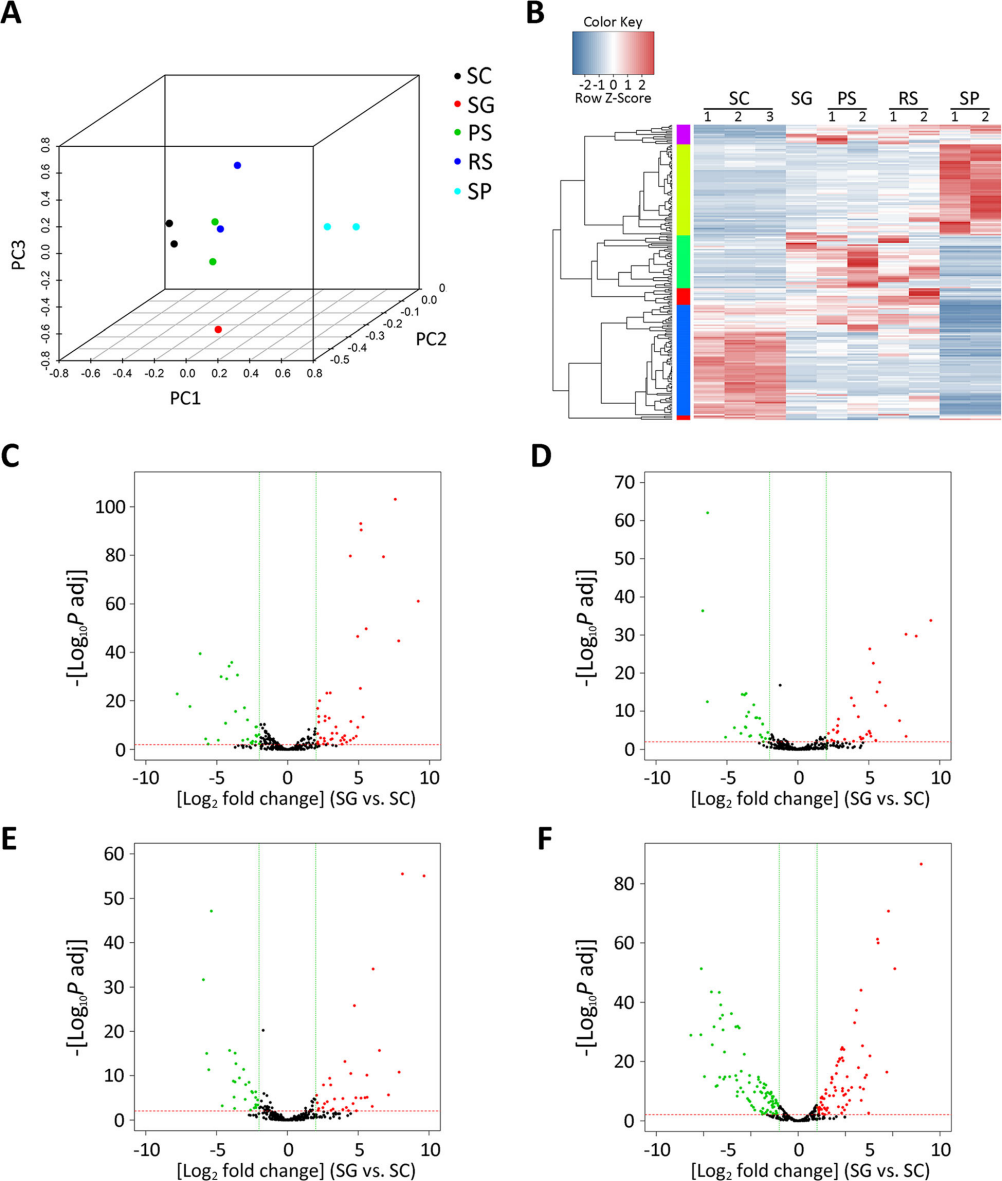
Analysis of miRNAs expression in SC, SG, PS, RS and sperm (SP). (**A**) Principal components explain the correlation among SC, SG, PS, RS, SP. (**B**) Heatmap comparing miRNAs’ expression among SC, SG, PS, RS and SP. (**C**) Scatter plot between SG and SC. (**D**) Scatter plot between PS and SC. (E) Scatter plot between RS and SC. (**F**) Scatter plot between RS and SC. Standard selection criteria to identify differentially expressed genes was established at *P*adj < 0.01; the log_2_ fold change > 2 were expressed as up genes (red dots), log_2_ fold change < − 2 were expressed as down genes (green dots)

To further analyze the expression pattern of miRNAs in SCs, we compared SC seq dada with small RNA data from germ cells (GSE124099) that was reported previously [26]. Principal component analysis (PCA) revealed that three SC samples clustered together (Fig. 2A), suggesting the reproducibility of the seq data. Notably, miRNA expression in SCs was different from that in germ cells (Fig. 2A and B, Additional file 2). Specifically, 27 miRNAs were high abundance in SCs and 47 miRNAs were abundant in spermatogonia (Fig. 2C, Additional file 2). The 26 and 35 miRNAs were identified specifically in SCs and pachytene spermatocytes, separately (Fig. 2D, Additional file 2). In addition, 28 miRNAs were enriched in SCs and 34 miRNAs were enriched in round spermatids (Fig. 2E, Additional file 2). Compared with ejaculated spermatozoa, 88 miRNAs were specifically in SCs and 71 miRNAs were specifically in spermatozoa (Fig. 2F, Additional file 2). That differentially enriched miRNAs between SCs and germ cells indicates the unique role of specific miRNAs in SCs and spermatogenesis.

### Prediction of the miRNA function

**Fig. 3:**
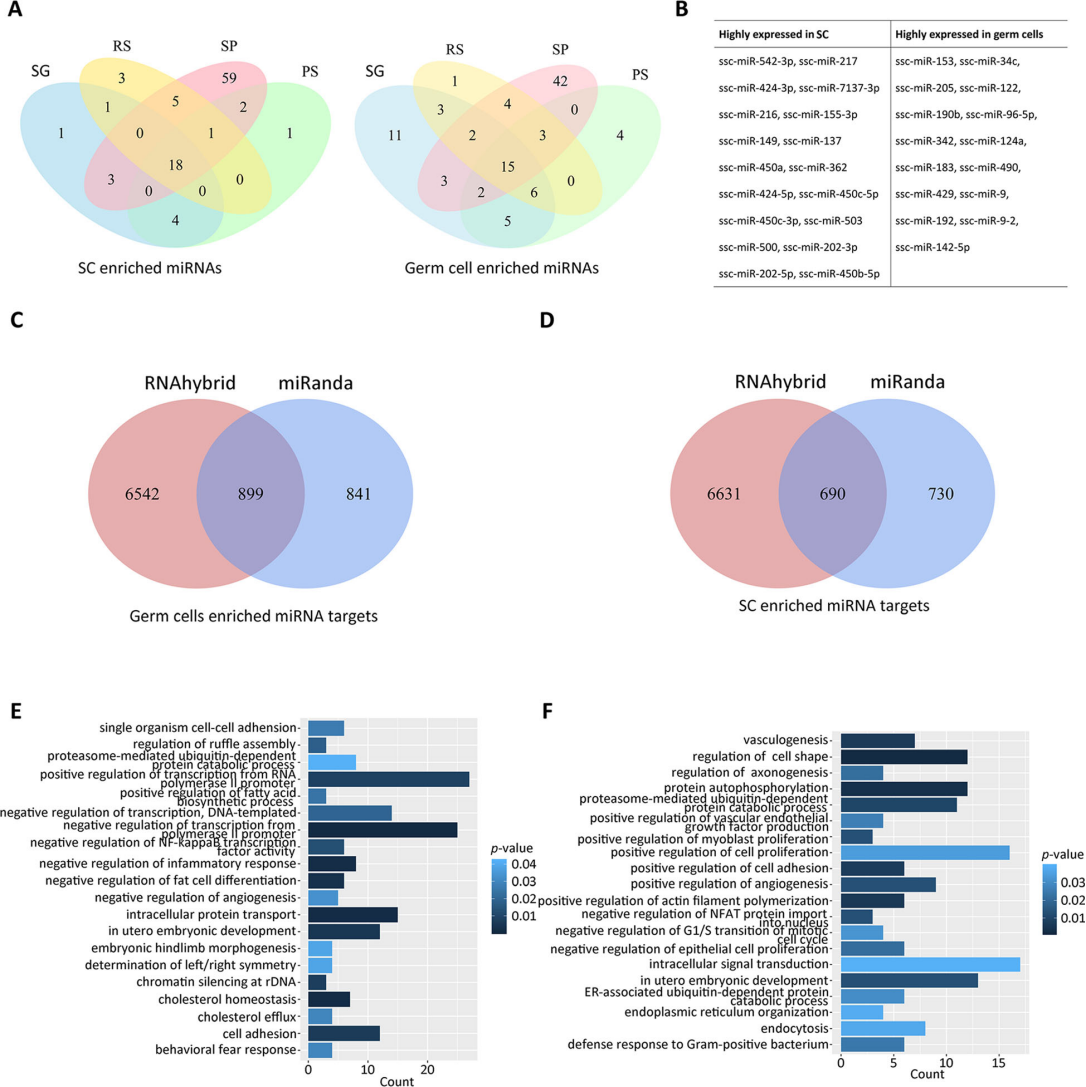
The function of MSC and germ cell enriched miRNAs. (**A**) Venn diagram of upregulated miRNAs in MSC (left) and downregulated miRNAs in SC (right). (**B**) List of highly expressed miRNAs in SC and germ cells. (**C**) Venn diagram of RNAhybrid predicted target genes and miRanda predicted target genes of germ cells enriched miRNA targets. (**D**) Venn diagram of RNAhybrid predicted target genes and miRanda predicted target genes of SC enriched miRNA targets. (**E**) GO analysis (biological process) of germ cells enriched miRNA targets. (**F**) GO analysis (biological process) of SC enriched miRNA targets

To obtain miRNA expression pattern in SCs and germ cells, we turn to identify the miRNAs enriched in SCs and germ cells. The Venn diagrams showed that 18 miRNAs were enriched in SCs compared with germ cells (Fig. 3A), indicating the high expression of these miRNAs in SCs (Fig. 3B). Furthermore, we found that 15 miRNAs were enriched in germ cells compared with SCs (Fig. 3A), which was considered as highly expressed in germ cells (Fig. 3B). miRNAs have been extensively reported to execute the functions through binding to their targets. To elucidate the biological function of miRNAs in SCs and spermatogenesis, miRNA targets were predicted by miRanda and RNAhybrid, respectively, which were considered as potential miRNA targets. The 899 miRNA targets for germ cells (Fig. 3C, Additional file 3) and 690 miRNA targets for Sertoli cells (Fig. 3D, Additional file 3) were used for gene ontology analysis. The results showed that germ cell enriched miRNAs were mainly involved in transcription, intracellular protein transport, cell adhesion (Fig. 3E). Interestingly, SC highly expressed miRNAs were related to cell shape, protein autophosphorylation, proteasome-mediated ubiquitin-dependent protein catabolic process, intracellular signal transduction, cell surface receptor signaling pathway, and positive regulation of cell proliferation (Fig. 3F).

### The potential roles of SC enriched miRNAs

**Fig. 4:**
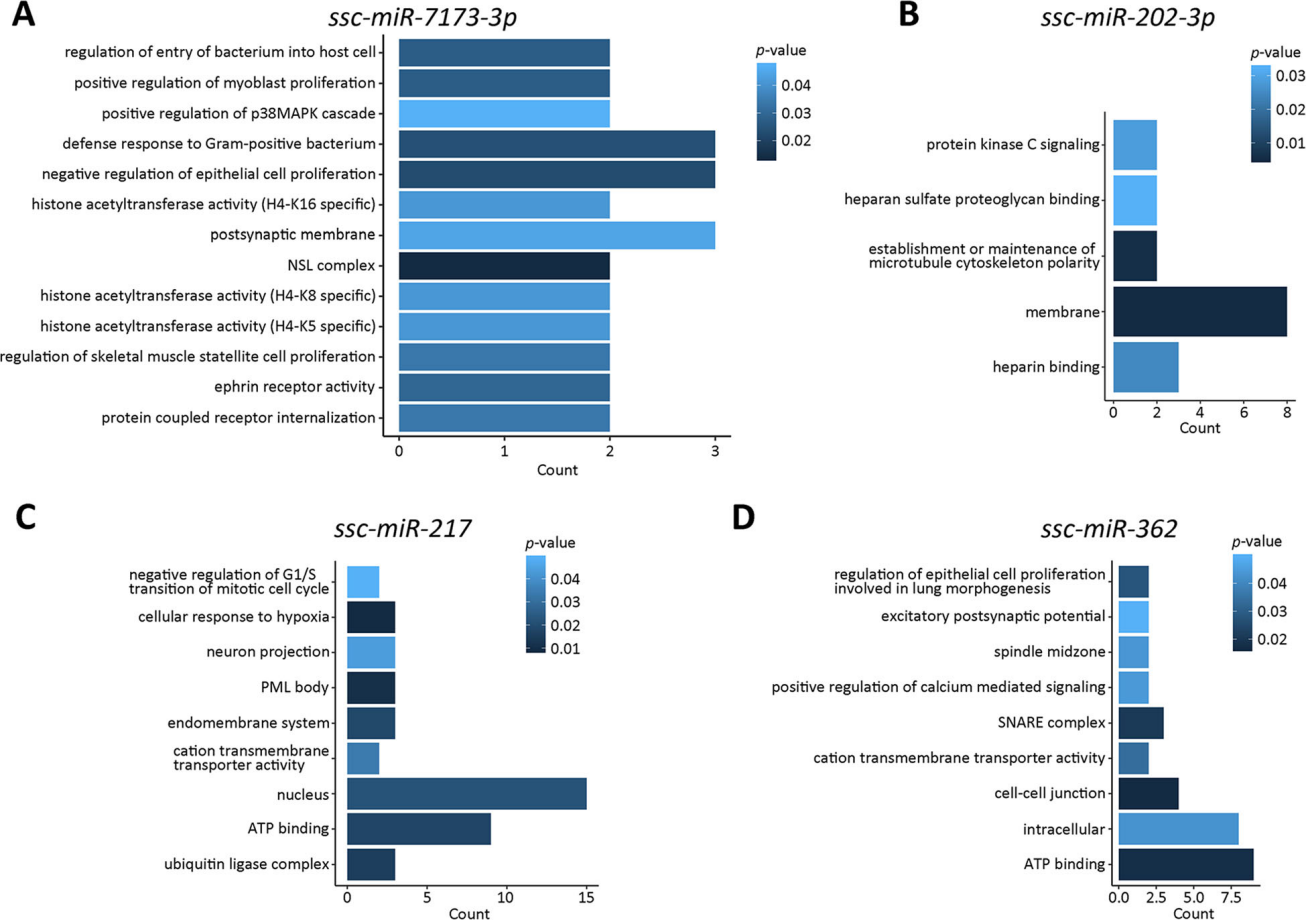
GO analysis of SC enriched miRNAs. (**A**) *ssc-miR-7173-3p* targets. (**B**) *ssc-miR-202-3p* targets. (**C**) *ssc-miR-217* targets. (**D**) *ssc-miR-362* targets

To identify the function of the SC enriched miRNAs, we conducted gene ontology analysis for each miRNA. Analysis revealed that there were 5 miRNAs exhibiting important functions. *Ssc-miR-7173-3p* participates in the epigenetic modification including histone acetyltransferase activity of H3K16, H3K8 and H3K5 (Fig. 4A), and s*sc-miR-202-3p* plays roles in regulation of protein kinase C signaling (Fig. 4B). In addition, s*sc-miR-217* negatively regulates G1/S transition in mitotic cell cycle (Fig. 4C), and *ssc-miR-362* functions in regulating cell-cell junctions (Fig. 4D).

**Fig. 5:**
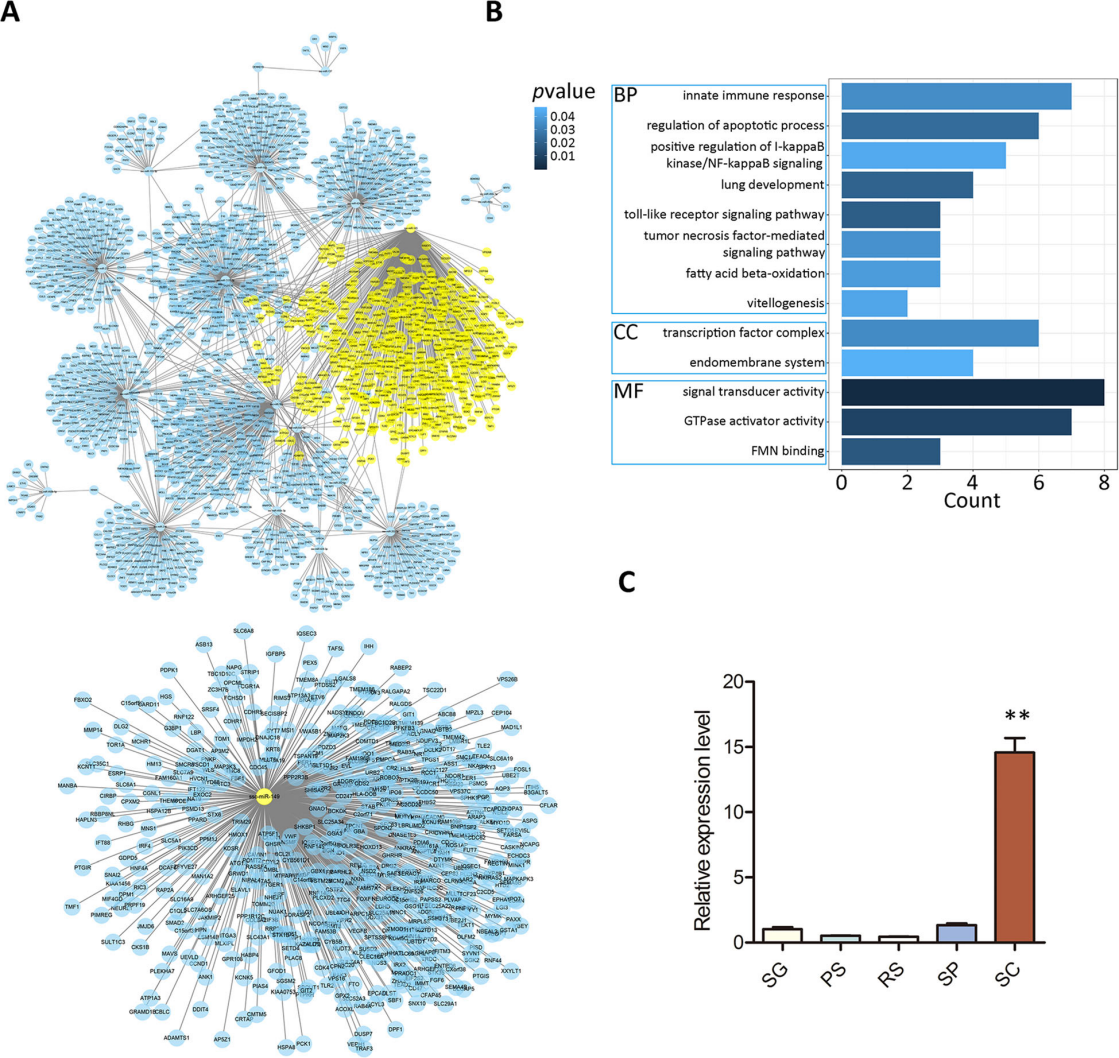
Function and expression pattern of *ssc-miR-149*. (**A**) Net plot of MSC enriched miRNA targets. The targets of *ssc-miR-149* were marked by yellow dot and showed in the lower panel. (**B**) GO analysis of *ssc-miR-149* targets. (**C**) qRT-PCR analysis of *ssc-miR-149* expression in SC, SG, PS, RS and SP. Data were presented as the mean ± SEM, ** *P* < 0.01

Specifically, a net plot generated by cytoscape revealed that *ssc-miR-149* possessed most miRNA targets compared with other miRNAs enriched in SCs, suggesting the vital role of *ssc-miR-149* in mediating SC functions (Fig. 5A, Additional file 4). Gene ontology analysis showed that *ssc-miR-149* participated in the biological processes of innate immune response, apoptotic process, and signaling pathway (I-kappa B knase/NF-kappaB signaling, toll-like receptor signaling and tumor necrosis factor-mediated signaling), and was located in the transcription factor complex and endomembrane system. Molecular function analysis revealed that *ssc-miR-149* mediated signal transducer activity, GTPase activator activity, and FMN binding (Fig. 5B). Importantly, the expression level of *ssc-miR-149* in SCs was 15-fold higher than that in other cell types (Fig. 5C). Hence, we supposed *ssc-miR-149* as an important miRNA in porcine SCs.

### *ssc-miR-149* targets tumor necrosis factor receptor (TNFR)-associated factor 3 (*TRAF3*)

**Fig. 6:**
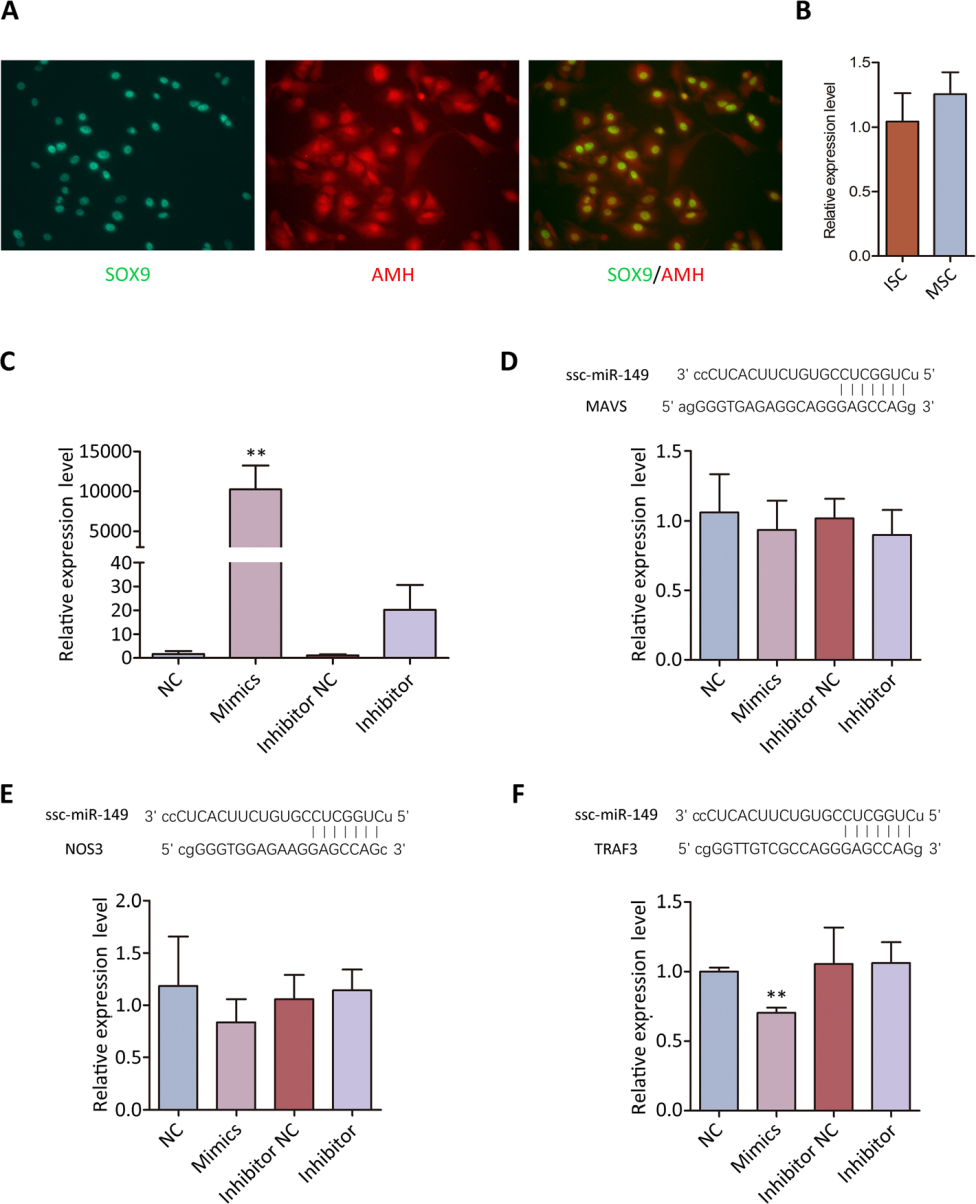
Analysis of the potential targets of *ssc-miR-149*. (**A**) Immunocytochemistry analysis of the isolated immature Sertoli cells (IMSC). (**B**) The expression pattern of *ssc-miR-149* in mature Sertoli cells (MSC) and immature Sertoli cells (ISC). (**C**) The expression level of s*sc-miR-149* in different treatments. (**D**) The expression level of *MAVS* in different treatments. (**E**) The expression level of *NOS3* in different treatments. (**F**) The expression level of *TRAF3* in different treatments. Data were presented as the mean ± SEM, ** *P* < 0.01

To determine whether *ssc-miR-149* expression changes during SC maturation, we isolated immature Sertoli cells (ISCs) that purity was determined and showed in Fig. 6A. Over 90% of the isolated immature SCs were positive for AMH and SOX9. qRT-PCR revealed that *ssc-miR-149* was consistently expressed during SC maturation (Fig. 6B). Therefore, we used immature SCs for further analysis. Transfection of *ssc-miR-149* mimics could significantly up-regulate *ssc-miR-149* expression (Fig. 6C). Interestingly, transfection of *ssc-miR-149* inhibitors also increased *ssc-miR-149* expression, suggesting the potential compensatory mechanism and the indispensable role of *ssc-miR-149* in SCs (Fig. 6C). Therefore, we focused on the regulatory roles of *ssc-miR-149*. Transfection of *ssc-miR-149* mimics had no effect on the expression level of mitochondrial antiviral signaling protein (*MAVS*, Fig. 6D) and nitric oxide synthase 3 (*NOS3*, Fig. 6E), but significantly down-regulated the expression level of *TRAF3* (Fig. 6F). In addition, transfection of *ssc-miR-149* inhibitors had no effect on the expression level of *MAVS*, *NOS3* and *TRAF3* (Fig. 6D to 6F), which might be due to the upregulation of *ssc-miR-149*.

**Fig. 7:**
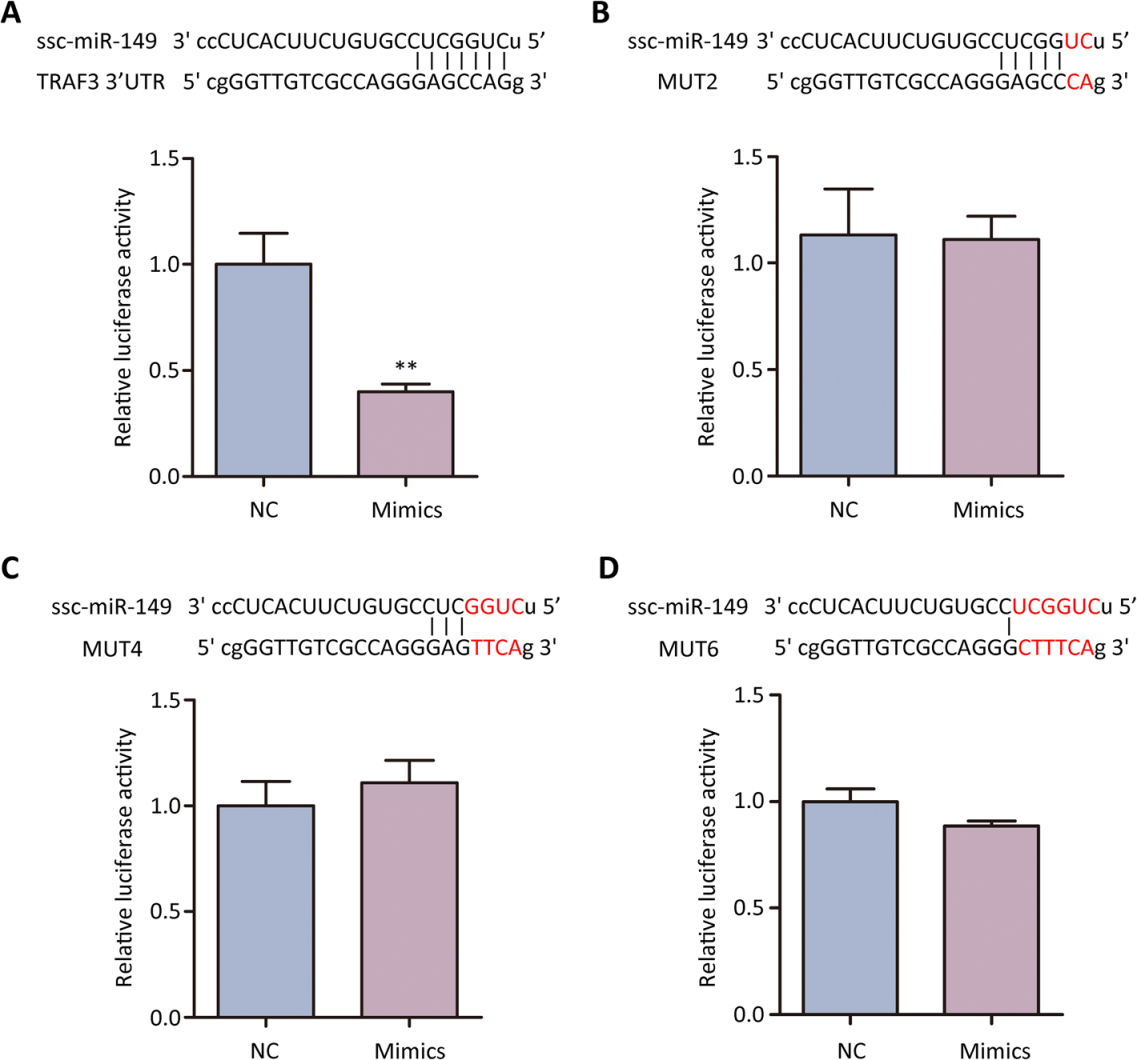
Validation of the target of *ssc-miR-149* by using dual luciferase assay. The validation of s*sc-miR-149* targets to wild type sequence of 3’UTR of *TRAF3* mRNA (**A**), with 2 mutation sites (**B**), 4 mutation sites (**C**), and 6 mutation sites (**D**). Data were presented as the mean ± SEM, ** *P* < 0.01

Next, we determined the potential relation between *ssc-miR-149* and *TRAF3*. *ssc-miR-149* mimics suppressed the luciferase activity of *TRAF3* 3’UTR (Fig. 7A). However, no effect was detected when the 2, 4, 6 sites of seed region in the targeting site of *ssc-miR-149* were mutated (Fig. 7C to 7D). Taken together, we demonstrated that *TRAF3* was a target of *ssc-miR-149*.

### *ssc-miR-149* disturbs the *TRAF3* downstream signaling pathway

**Fig. 8:**
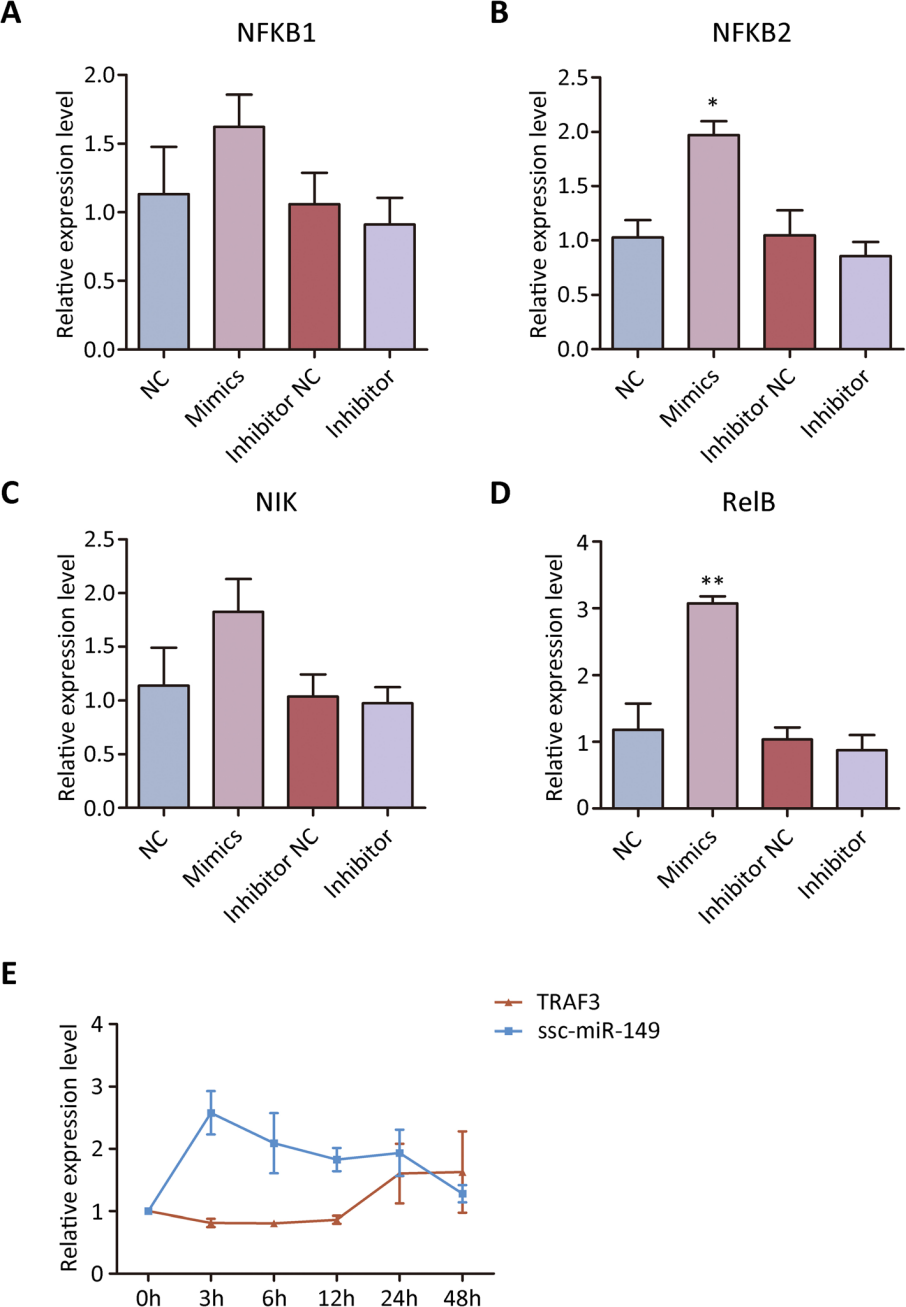
The expression of *TRAF3* downstream signaling pathway genes. The expression level of *NFKB1* (**A**), *NFKB2* (**B**), *NIK* (**C**), *RelB* (**D**) among different treatments. The expression level of *ssc-miR-149* and *TRAF3* in SCs after LPS challenges (**E**)

It has been reported that *TRAF3* controlled lymphotoxin beta receptor (LTBR)-dependent activation of both the canonical and non-canonical nuclear factor kappa B (NFKB) pathways, and that knock-down of *TRAF3* increased nuclear factor kappa B subunit 2 (*NFKB2*), RelB proto-oncogene (*RelB*) and NF-kappa-B-inducing kinase (*NIK*) expression [29]. We further analyzed whether overexpression of *ssc-miR-149* could disturb *TRAF3* downstream gene expression. Analysis revealed that transfection of *ssc-miR-149* did not affect the expression of nuclear factor kappa B subunit 1 (*NFKB1*) (Fig. 8A), but significantly up-regulated *NFKB2* expression (Fig. 8B). In addition, NIK expression remained in the regular level (Fig. 8C), and *RelB* expression was up-regulated (Fig. 8D). Again, transfection of *ssc-miR-149* inhibitor did not affect the expression of *NFKB1*, *NFKB2*, *NIK*, and *RelB* (Fig. 8A to 8D). Together, *ssc-miR-149* could mediate *NFKB2* and *RelB* expression through targeting *TRAF3*.

### Time course effects of LPS treatment on *ssc-miR-149* and *TRAF3* expression

LPS that is known to induce the inflammatory response was reported to reduce the growth of SCs [30]. To determine the effect of LPS treatment on expression of *ssc-miR-149* and *TRAF3*, the SCs were treated with 10 μg/mL LPS as previously reported [30]. The result showed that LPS could induce *ssc-miR-149* expression in a time dependent manner (Fig. 8E). Meanwhile, the expression level of *TRAF3* exhibited high association with *ssc-miR-149* expression (Fig. 8E), indicating that *ssc-miR-149* targets *TRAF3* and mediates SC immune response.

## Discussion

Previous studies have demonstrated the crucial functions of SC miRNAs including control of proliferation, maturation, BTB, and the stage of seminiferous epithelium cycle [16]. In the present study, we found that half of the small RNAs in porcine SCs were miRNAs, suggesting the potential roles of miRNAs in regulation of SC functions. miRNAs act as a powerful regulator to control a wide range of biological processes, and are highly, exclusively and preferentially expressed in testicular cell types [31]. Previous studies have identified a series of differentially expressed miRNAs in mature and immature testis, or in different testicular cell population via high throughput sequencing [32]. Although miRNAs profiles of the SCs were assessed through sequencing, microarray and qPCR, systematical analysis of SC miRNAs is required. Therefore, we profiled miRNAs expression by comparing the miRNAs in testicular germ cells and in SCs. Analysis revealed that 18 miRNAs were abundant in SCs and 15 miRNAs were testicular germ cell specific. Because expression of androgen receptor is not shut down after 48 h culture of Sertoli cells [33], the screened miRNAs were little or no associated with androgen receptor, and the relationship between miRNAs expression and androgen receptor expression is worthy for future determination. Interestingly, *miR-202-5p/3p* was reported to be expressed in a sexually dimorphic pattern as the primordial XY gonad differentiates into a testis, with strong expression in SCs, suggesting a potential role in early testis development [34]. Moreover, *miR-202-5p/3p* also participate in pathogenesis of azoospermia or Sertoli-cell-only syndrome [35]. In addition, *miR-34c* that was highly enriched in porcine germ cells has been demonstrated to function in spermatogenesis and early embryo development [36]. Hence, we aimed the highly enriched miRNAs in SCs versus in germ cells. It makes sense that future study needs to explore the other miRNAs that highly enriched in germ cells versus in SCs.

MiRNAs function as a guide by base pairing with their target mRNA to mediate the binding of AGO proteins, inducing translational repression, mRNA de-adenylation and mRNA decay [37]. Particularly, the base 2–8 at the 5’end of miRNAs, known as seed region, is crucial for target recognition of mRNA in the 3’UTR [37]. Therefore, it is not surprising that a single miRNA has hundreds of targets and a mRNA target possess multiple binding sites for the same or different miRNAs. Here, 899 mRNAs and 690 mRNAs were predicted as germ cell enriched miRNA targets and SC enriched miRNA targets, respectively. Gene ontology analysis revealed that germ cells enriched miRNAs regulate transcription, intracellular protein transport, cell adhesion. Normal progression of spermatogenesis requires accurate and spatiotemporal regulation of gene expression [38]. The regulation at the transcriptional level indicates the sequential and coordinated regulatory roles of miRNAs in spermatogenesis [39]. The progressive transformation of SSCs into highly specialized spermatozoa is controlled by intrinsic or extrinsic signals [40]. The intrinsic factor (e.g., transcriptional factors) movement between the cytoplasm and nucleus dictates germline cell differentiation [41], suggesting that miRNAs could mediate the germ cell differentiation through controlling the intracellular protein transport. Furthermore, the spermatozoa appropriately releasing from SCs is dependent on cell adhesion [42]. The findings implied the potential roles of miRNAs in controlling the release of spermatozoa.

Notably, analysis revealed that the highly expressed miRNAs in SCs participated in cell shape, protein autophosphorylation, proteasome-mediated ubiquitin-dependent protein catabolic process, intracellular signal transduction, cell surface receptor signaling pathway, and positive regulation of cell proliferation. SCs are one of the most complicated, three-dimensional structures in cell biology [5]. They own the long cytoplasmic arms to form cup-like areas to hold and nurture the germ cells [5]. On account of their large plasma membrane (16,000 μm^2^), SCs are involved in the translocation of numerous organelles, the metabolization of hundreds of different classes of proteins for specific functions, the transport of protein to specific region throughout the cycle of seminiferous epithelium [5, 43–45]. The complicated functions of SCs need coordinated regulation of multiple regulatory pathways. SCs serve as the target of hormone (FSH and testosterone) to transduce the endocrine signals and other cellular cues into germ cells [46]. However, the response of SC to hormone stimulation varies with testis development and with the stage of seminiferous epithelia [5]. Considering that SC maturation is regulated by androgen and FSH [15], it is worth of further analyzing miRNA dynamics during porcine SC maturation. The findings of miRNA function in biological processes would provide references for comprehending the numerous functional interactions and regulatory networks in SCs.

To explore the important miRNAs, the miRNA targets were analyzed by cytoscape. Analysis revealed that *ssc-miR-149* possesses the most potential target genes, suggesting the crucial roles of *ssc-miR-149* in SCs. The further analysis showed that *ssc-miR-149* mediates innate immune response, immunology signaling pathway, and apoptotic processes. The BTB formed by SCs provides the unique immunoregulatory environment for germ cells [9]. In addition to immune privilege environment, SCs could also express apoptosis inhibitors, complement inhibitors, immunomodulatory factors, anti-inflammatory cytokines and chemokines to mediate the immune response and protection of germ cells [10, 47, 48]. The roles of *ssc-miR-149* provide the evidence that miRNAs might regulate the SC mediated immune privilege.

We found that ssc-*miR-149* targeted *TRAF3*. *TRAF3*, a member of *TRAF* family, is one of the most multi-functional *TRAF* molecules [49]. Accumulating data have demonstrated that *TRAF* family not only participates in the signaling of TNFR family, but also participates in the innate immune receptors, the Toll-like receptor (TLR) family, nucleotide binding-oligomerization domain-like receptors (NLR) and the retinoic acid-inducible gene (RIG)-I-like receptor family [50–52]. It has been reported that SCs play roles in the testicular antiviral defense system and bactericidal testicular defense mechanism mediated by panels of TLRs and RIG-I [53, 54], indicating the potential roles of TRAF3 in mediating immune response in SCs. TRAF3 negatively regulates LTBR signaling via both canonical and non-canonical NFKB pathways, and inhibits the expressions of *NFKB3*, *RelB,* and *NIK* [35]. Downregulation of *TRAF3* by *ssc-miR-149* induced the upregulation of *NFKB2* and *RelB*, which further demonstrated the roles of *ssc-miR-149* in the immune system. Hence, future studies are needed to identify *ssc-miR-149* function in mediating the initiation of immune response in SCs.

In addition to miRNAs, we found that there were small proportion of tsRNAs and rsRNA in SCs. The tsRNAs has been demonstrated to regulate the cellular response to the environmental stress [55]. Also, tsRNAs act as acquired epigenetic factors in mouse spermatozoa to reflect the low protein diet and high fat diet stress, which mediate intergenerational inheritance [56, 57]. Interestingly, we found that semen-derived exosomes delivered tsRNAs to porcine spermatozoa. By microinjection of antisense sequences into *in vitro* fertilized oocytes and subsequent single-cell RNA-seq, we identified a specific functional tsRNA group that participate in the early cleavage of porcine preimplantation embryos, probably by regulating cell cycle-associated genes and retrotransposons (unpublished data). Thus, specific tsRNAs presented in porcine spermatozoa play significant roles in preimplantation embryo development. In addition, rsRNAs were involved in the acute phase of mouse body inflammation [58]. Therefore, the existence of tsRNAs and rsRNAs in porcine SCs would provide a new field to study small RNA functions in supporting spermatogenesis.

## Conclusion

We identified a subset of SC highly enriched miRNAs and revealed their potential roles in SCs. Further analysis found that *ssc-miR-149* mediated immune response through targeting TRAF3. The findings of this study would facilitate the study on SC functions.

## Supplementary information

**Additional file 1.** List of primers used in this study.

**Additional file 2.** List of differentially expressed miRNAs.

**Additional file 3.** List of SC enriched or germ cell enriched miRNA targets.

**Additional file 4.** List of SC enriched miRNA targets.

## Data Availability

All data supporting our findings are included in the manuscript.

## Abbreviations

BMP4: Bone morphogenetic protein 4
BTB: Blood-testis barrier
LPS: Lipopolysaccharide
LTBR: Lymphotoxin beta receptor
MAVS: Mitochondrial antiviral signaling protein
miRNAs: MicroRNAs
NFKB1: Nuclear factor kappa B subunit 1
NFKB2: Nuclear factor kappa B subunit 2
NIK: NF-kappa-B-inducing kinase
NLR: Nucleotide binding-oligomerization domain-like receptors
NOS3: Nitric oxide synthase 3
PAK2: P21 activated kinase 2
PBS: Phosphate buffered saline
PCA: Principal component analysis
RA: Retinoic acid
RelB: RelB proto-oncogene
RIG: Retinoic acid-inducible gene
SCF: Stem cell factor
SCs: Sertoli cells
SPAG1: Sperm associated antigen 1
SSCs: Spermatogonial stem cells
TLR: Toll-like receptor
TRAF3: Tumor necrosis factor receptor (TNFR)-associated factor 3
